# Supplementary figures and images for: Evaluation of hematological parameters alterations in different waves of COVID-19 pandemic: A cross-sectional study
Source: PLoS One. 2023 Aug 25;18(8):e0290242. doi: 10.1371/journal.pone.0290242 (PMC10456189; doi:10.1371/journal.pone.0290242)

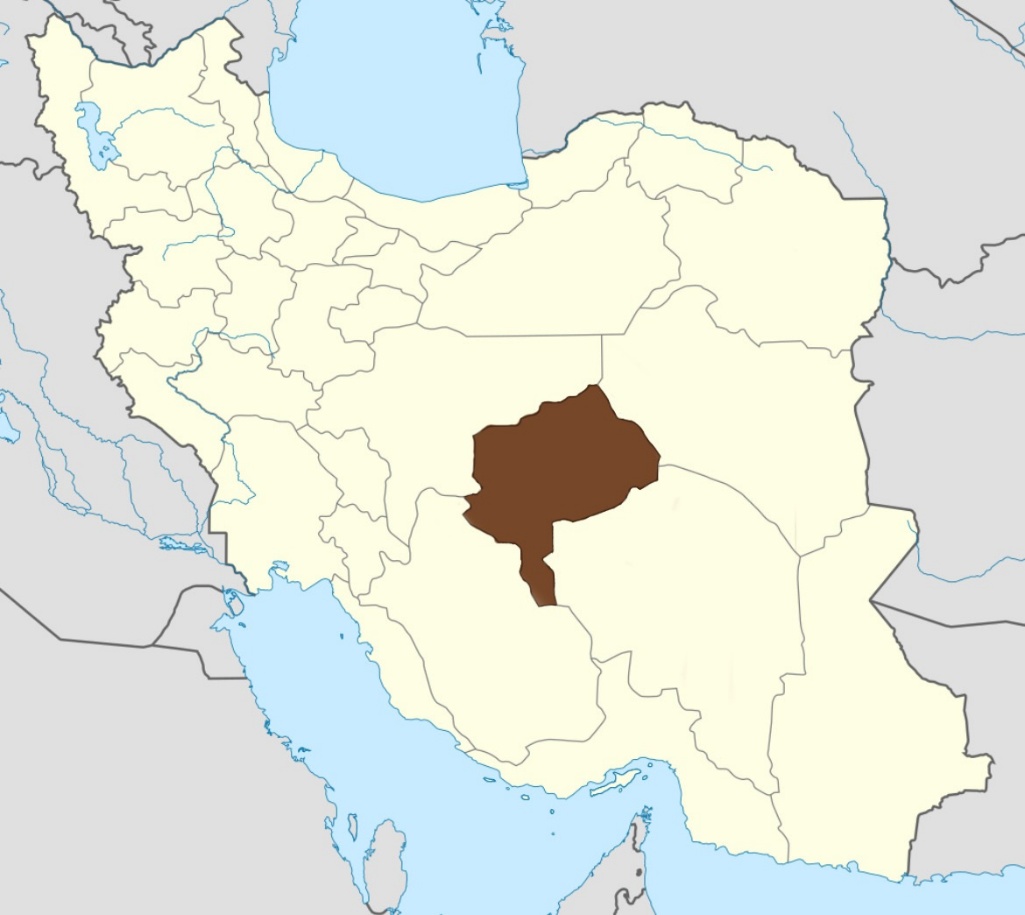


Figure Supplementary. The geographical position of the province of Yazd in Iran.

Supplement: S1 File — (DOCX) [file pone.0290242.s001.docx]
