# Supplementary material for: Evaluation of hematological parameters alterations in different waves of COVID-19 pandemic: A cross-sectional study
Source: PLoS One. 2023 Aug 25;18(8):e0290242. doi: 10.1371/journal.pone.0290242 (PMC10456189; doi:10.1371/journal.pone.0290242)
Supplement: Attachment — Submitted filename: Response to reviewers.docx [file pone.0290242.s002.docx]

**We greatly appreciate the effort and time you have dedicated to providing valuable feedback on our manuscript. We are grateful to the reviewers for their insightful comments on my paper. We have been able to incorporate changes to reflect most of the suggestions provided by the reviewers.**

Reviewer #1:

This paper looks at hematological parameters in different waves, by recruiting patients at the hospital. By definition these people are not healthy and a control (COVID negative) is required at each wave.

Response: thanks for your valuable suggestion. The current research was performed in the main COVID-19 center hospital, Shahid sadoughi hospital, affiliated with Yazd University of Medical Sciences, in Yazd province, that at that time, the focus was on admitting people with COVID-19 and all the departments of the hospital were assigned to this positive group, so unfortunately non-covid-19 people were not admitted to this hospital.

We have no information on the waves (viral strain, evaluated infection levels in the general population) that renders the information un-informative.

Response: Considering that these data were collected from the city of Yazd and reached us, except in the third wave, which was the type of virus variant x, more information about the type of virus variant in the rest of the waves and the level of involvement of the general Population in the form of documents not available.

These type of studies need multiparametric studies. There are too many confusing factors (not only age and sex).

Response: Thank you for your valuable point. Since the data collection time was at the peak of the COVID-19 and at that time our data source was the files extracted from the Hospital information system, the variable items and existing confusing factors were limited for the entire study population.

Reviewer #2:

1. On occasion, spelling and verbiage is used inconsistently. I would encourage the authors to revise their manuscript for consistency. For example,

- While covid-19 mainly affects the respiratory system […], So far several models for the prognosis of the disease of COVID-19 […], Numerous reports of the disorder of hematological parameters levels in Covid-19 patients […]

- […] the body is another target of this wild virus. It is unclear what the authors signify by “wild virus”.

- Some words are unnecessarily capitalized (hematological Parameters; and There is an urgent; And Also, from the early detection; Quantitative Characteristics of patients)

Response: Thank you for your valuable point. All the items that were correctly mentioned have been corrected.

2. Some statements made by the authors should be substantiated with references. I would encourage the authors to review the current literature to better position their own study and findings. For example,

- So far, several models for the prognosis for the disease of COVID-19 have been reported. What are these models?

Response: According to PMID: **32265220** study, various models have been made for this prediction, which was added to the text references at your suggestion.

- A study conducted in five different countries in Asia, Africa, and the United States shows that CBB is the most common laboratory test. These studies should be cited.

Response: According to PMID: **30535132** study, which was added to the text references at your suggestion.

- Numerous reports of the disorder of hematological parameters levels in Covid-19 patients have been mentioned in various studies, some of which had a significant relationship with the clinical status and prognosis of the patients. It’s unclear which studies are being referenced here.

Response: According to PMID: **30535132** study, which was added to the text references at your suggestion.

- Previous studies showed that leukocytosis predicts a poor prognosis in critically ill Covid-19 patients. Which studies?

Response: According to PMID: **32822430,** PMID: **32533986, and** PMID: **34126954** studies, several studies showed that leukocytosis predicts a poor prognosis, which were added to the text references at your suggestion.

3. The authors should provide a justification for their sample size. Why did they only select n=300 patients per wave of COVID-19? The authors potentially have access to a much larger dataset and this could strengthen their findings. Also, the authors should provide a justification as to why they chose not to include asymptomatic controls/negative for comparison.

Response: G-Power software was used to calculate the sample size. Considering the effect size of 0.09, the alpha error of 0.05, the power of the test 0.80, and the number of groups equal to 5, the sample size was calculated as 1480, as a result, approximately 300 samples were needed for each group.

Below you can see the output of this software along with the alpha and beta error graph.

**F tests -** ANOVA: Fixed effects, omnibus, one-way

**Analysis:**	A priori: Compute required sample size

**Input:**	Effect size f	=	0.09

α err prob	=	0.05

Power (1-β err prob)	=	0.80

Number of groups	=	5

**Output:**	Noncentrality parameter λ	=	11.9880000

Critical F	=	2.3779612

Numerator df	=	4

Denominator df	=	1475

Total sample size	=	1480

4. In general, how much time was there between a positive RT-PCR test and hematological assessment in the patient population? Was this similar in all three categories of patients (i.e., partially recovered, recovered, and deceased)? How would delays between a positive RT-PCR and hematological assessment affect the study’s findings? Sampling delays could significantly bias the study’s findings and should be acknowledged by the authors.

Response: Thanks for your appropriate point. All PCR tests were taken for all people (Referred individuals with symptoms) in all groups at the same time and upon their arrival at the hospital. The cases with PCR positive result (Up to 24 hours) were subjected to the evaluation of hematological parameters. This item mentioned in the manuscript “Study design and participants” and highlighted.

5. Some acronyms are not defined (ex: MCH, MCHC, MCV, PDW, PLT). While these acronyms might be common in hematology, PLoS One’s broad readership would benefit from additional definitions.

Response: Thank you for your valuable point. All the items that were correctly mentioned have been corrected.

6. The distribution of patients aged < and ≥ 65 years […]. Is there a value missing after the “<”?

Response: Thank you for your valuable point. All the items that were correctly mentioned have been corrected.

7. The authors should provide a definition for “partial recovery” and “recovery”. The difference between these two categories is not clear.

Response: Partial recovery refers to people who, although the process of improving their condition was observed, but they had not fully recovered and they needed to continue the treatment, so that either anti-COVID-19 medication should be received at home or, if necessary, they would go to the doctor's office or hospital to follow up their condition. Recovery refers to patients were those who were completely treated and did not need to go to the doctor or hospital again.

8. It is difficult to appreciate the study’s findings because continuous data is being presented in a Table versus Figures. I would highly recommend that the authors use Figures present the data found in Tables 2-5. Currently, it is difficult to appreciate changes in hematological parameters from the 1st to 5th waves of COVID-19, age groups, sex, and disease severity. In addition, it is not very clear from the tables where these significant changes are happening. I would recommend the authors use box-and-whisker plots (where applicable) to present their findings. Using figures would also complement the text.

Response: Thank you for your valuable point. All the items that were correctly mentioned have been corrected and revealed in box-and-whisker plot.

9. As stated by the authors, the findings from this study could be useful for optimizing clinical decisions in countries that suffer from significant shortages of medical resources, potentially reducing mortality rates. The authors show that hematological parameters vary from wave to wave of COVID-19. However, they do not discuss how these changes affect the association of hematological parameters and disease severity. Do we see a change in sensitivity and specificity from wave to wave? The authors should report sensitivity and specificity data to (1) allow comparisons with other similar studies and (2) allow the reader to assess the usefulness of using hematological parameters for disease prognosis.

Response: If we have understood your meaning correctly, our answer to you is: you ask a good question, as the science of epidemiology shows us, the prevalence of a disease has an effect on the sensitivity and specificity of its diagnostic test. However, in this study, we did not seek to investigate the prevalence and only studied the patients with covid-19, who were already confirmed to be sick with a gold standard (PCR).

10. The authors specify that “those patients who had missing data in their hematological parameters […] were negatively excluded” but the N= reported in each table would suggest otherwise. For example, in Table 3 N=716 values are reported for hematocrit while N=687 values are being reported for lymphocyte. Shouldn’t each parameter have an equal number of patients? Table 1 suggests that 717 patients <65 were included in this study, but Table 3 reports 716 hematocrit measurements, 666 neutrophil measurements, etc. Some further explanation would be valuable here. The authors should report the sample size (n=), denominators (N=), and proportions (%) for each hematological parameter in each Table to facilitate interpretation. A flow-chart could also assist the reader in understanding how patients were selected, included, excluded, and which hematological parameters were available at the time of the study.

Response: Thank you for your valuable point. All the items that were correctly mentioned have been corrected in tables. Due to the limitations of the laboratory and personnel workload, all tests are not performed routinely in this center, and the reason for the difference between the level of lymphocytes and the hematocrit is due to this.

Reviewer #3:

1. I suggest the authors should add a quick description of the study city or site for international readers

Response: Thank you for your valuable point. All the items that were correctly mentioned have been corrected.

2. Throughout the main text, the authors emphasized on different waves of Covid-19 in Iran but it is not clear for the reader when and for how long these waves occurred. I therefore recommend the authors to present a figure showing different waves of Covid-19 in Iran.

Response: Thank you for your valuable point. All the items that were correctly mentioned have been corrected and mentioned in figure 2.

3. There are a number of grammatical mistakes and abbreviations in the text without any initial explanations during the first appearance. Authors should correct that.

Response: Thank you for your valuable point. All the items that were correctly mentioned have been corrected.

4. Some unnecessarily long sentences should also be broken into at least two.

Response: Thank you for your valuable point. All the items that were correctly mentioned have been corrected.
