# Supplementary material for: Evaluation of hematological parameters alterations in different waves of COVID-19 pandemic: A cross-sectional study
Source: PLoS One. 2023 Aug 25;18(8):e0290242. doi: 10.1371/journal.pone.0290242 (PMC10456189; doi:10.1371/journal.pone.0290242)
Supplement: Attachment — Submitted filename: response.docx [file pone.0290242.s003.docx]

**We deeply value the dedication and time you have invested in offering valuable feedback on our manuscript. We extend our sincere gratitude to the reviewers for their perceptive comments on our paper. We have successfully integrated modifications to address the majority of the suggestions put forth by the reviewers.**

**Reviewer #1:**

**Q:** The authors did not respond to any of my comments.
My comments remain, and I believe that the data provided is not sufficient for publication.

**A:** Thank you for your comment. We have made sincere efforts to incorporate the changes based on your feedback, and we greatly appreciate your detailed comments. However, it is important to acknowledge that some limitations exist within our study, and we have addressed these limitations to the best of our abilities. The research which conducted at Shahid Sadoughi Hospital, the main COVID-19 center affiliated with Yazd University of Medical Sciences in Yazd province. The current study employed a cross-sectional approach, as evident from the main title of the manuscript. The primary aim of the study was to offer a comprehensive overview of different hematological parameters in individuals who tested positive for COVID-19 throughout various waves, ranging from the first wave to the fifth wave. The main emphasis was placed on describing these parameters and presenting the findings obtained from the descriptive analysis conducted. Additionally, due to the limitations of routine hospital tests in detecting the specific variant type of the SARS-CoV-2 virus, the determination of variant types for each wave in our study was carried out by the Ministry of Health in our country. In order to accurately identify the variant, sequencing is required, which was not performed during the analysis to ensure the accuracy of the results. Therefore, the analyses conducted in our study were not based on the specific variant types. The data collection process occurred during the peak of the COVID-19 pandemic, where our primary data source consisted of files extracted from the Hospital Information System. Consequently, the study was limited by the availability of variable items and other confounding factors for the entire study population.

**Reviewer #2:** While the current manuscript is a significant improvement compared to the previous version, it requires additional revision to be considered for publication in PLoS ONE in my personal opinion.

**Q:** The authors still have not provided a justification as to why they chose not to include asymptomatic/negative/healthy controls for comparison.

**A:** Thank you for your valuable comment. The present study utilized a cross-sectional approach, as indicated in the main title of the manuscript. Our primary objective was to provide a general overview of various hematological parameters specifically among COVID-19-positive patients across different waves (from the first to the fifth wave). The main focus was on describing these parameters and reporting the findings resulting from this descriptive analysis.

**Q:** The manuscript is missing several references. For example:

[…] and their reduction can be extremely harmful to infection such as COVID-19 (ref).

[…] and their decreased number are less affected by infection (ref).

I would encourage the authors to carefully revise their manuscript for accuracy and completeness.

**A:** Thank you very much for your feedback. We appreciate your acknowledgement that our comment has enhanced the value of the manuscript. We have taken great care in revising and correcting all the mentioned items accordingly.

3. Several typographical errors are present in the manuscript, including in the figures. I would encourage the authors to carefully refine their manuscript. For example:

Figure 3, X-axis, “RDW_CV”, 2th (should be 2nd), 3th (should be 3rd), 4th and 5th

Table 1. […] with COVID-19 during the covid-19 pandemic

**A:** Thank you very much for your feedback. We appreciate your acknowledgement that our comment has enhanced the value of the manuscript. We have taken great care in revising and correcting all the mentioned items accordingly.

**Q:** The authors note in the results that there was a significant association between hematological parameters amongst “younger” and “elderly” patients. More specifically, the mean of WBC, neutrophil, and ESR were significantly higher in the “elderly” than in the “younger ones”. The level of hematocrit, hemoglobin, RBC, and ESR parameters were also significantly higher in men than in women, while the median level of lymphocyte and platelets were lower in men versus women. In addition, the distribution of patient age varies between the different waves of the COVID-19 pandemic (Table 1). However, aggregate data is presented in Figure 3. A stratified analysis based on different age categories would be helpful here. It is unclear if the differences observed are due to the different SARS-CoV-2 variants or the changing mean age of the patient population from wave-to-wave.

**A:** Thank you for your response. We appreciate your recognition of the value our comment has added to the manuscript. We understand that our study encountered limitations, one of which was the unavailability of variant strain information for individual waves. Consequently, the study focused solely on investigating age and gender, and the reported results are based on these variables. It is important to acknowledge and address these limitations within the study.

**Reviewer #3:** I'd that the authors for their effort to address the reviewers' comments to improve the overall quality of their manuscript.
The figure 2 showing the time frame of each of the covid-19 waves in Iran and its relationship with the patients outcome is particularly appreciated.
However, I still believe that one of my major comments below was not addressed.
Major points
1. I suggest the authors should add a quick description of the study city or site for international readers
This is important for international reader to better understand the epidemiological pattern of COVID-19 in the city of Yazd.
I recommend the authors to add a study site description section together with a map of Iran for more illustration.

**A:** Thank you very much for your feedback. We appreciate your acknowledgement that our comment has enhanced the value of the manuscript. We have taken great care in revising and correcting all the mentioned items accordingly. Additionally, a map form Yazd city has been added as a supplementary figure. Moreover, this paragraph has been added to article:

“Yazd city, is the capital of Yazd province the Central geographic region in Iran, and is recognized as a World Heritage Site by UNESCO [1]. Yazd has a hot desert climate and is known as the driest major city in Iran. Yearly precipitation amount in this town is less than 50 millimeters with summer temperatures very frequently above 40 °C (104 °F) in blazing sunshine with no humidity. Shahid Sadoughi University of Medical Sciences (SSU) is the only public medical university in this province and Shahid Sadoughi Hospital is the main affiliated hospital with SSU.”

1. Rezaee M, Charrahi Z. Spatial Planning and Tourism Development with Sustainability Model of the Territorial Tourist with Land Use Approach. Int J Soc Bus Sci. 2020;14: 1007–1012.
