# Supplementary material for: Evaluation of hematological parameters alterations in different waves of COVID-19 pandemic: A cross-sectional study
Source: PLoS One. 2023 Aug 25;18(8):e0290242. doi: 10.1371/journal.pone.0290242 (PMC10456189; doi:10.1371/journal.pone.0290242)
Supplement: Attachment — Submitted filename: Response to reviewers-19-JULY-R3.docx [file pone.0290242.s004.docx]

**We greatly appreciate the effort and time you have dedicated to providing valuable feedback on our manuscript. We are grateful to the reviewers for their insightful comments on my paper. We have been able to incorporate changes to reflect most of the suggestions provided by the reviewers.**

**Reviewer #2:**

**Q:** Typographical errors are still prevalent in the manuscript, including within the figures and tables. I would encourage the authors to revise their manuscript carefully.

**A:** We sincerely appreciate the time and effort you dedicated to reviewing our manuscript. Your insightful comments and constructive feedback have been immensely valuable in improving the quality of our research. We would like to express our gratitude specifically for pointing out the typographical errors present in the original version of the manuscript, including those within the figures and tables. We have taken your feedback seriously and conducted a thorough revision to address these issues diligently. During the revision process, we carefully corrected all the typographical errors in the main text, figures, and tables. Additionally, we have highlighted all the changes made to facilitate a clear understanding of the revisions. Moreover, based on the feedback received and our latest data, we have revised the content accordingly, aiming to enhance the accuracy and clarity of our findings. We hope that our efforts in revising the manuscript have met your expectations. Should you require any further information or have any additional concerns, we would be more than happy to address them promptly.

**Q:** I strongly encourage the authors to reconsider the conclusions they have drawn from their statistical analysis. Specifically, “Among the different waves of the pandemic, we observed that the third wave, associated with the B.1.1.413 variant originating from Western countries, Australia, and Canada, had the most significant impact in our observations”. As mentioned by the authors, mean ages and outcomes (partial recovery, full recovery, and death) varied significantly from wave to wave, therefore it is difficult to determine whether changes in hematological parameters were a result of the “third wave” or simply due to an older patient population and/or a higher proportion of deceased patients, without further statistical analysis.

**A:** We acknowledge and understand your concern regarding the conclusions drawn from our statistical analysis, particularly in regard to the impact of the B.1.1.413 variant and the higher mortality observed in the third wave of the pandemic. Your valuable comment prompted us to reevaluate our approach, and we wholeheartedly agree with your suggestion to refrain from attributing causation without conducting further statistical analysis. Upon careful consideration, we have made significant revisions to our manuscript to better clarify our findings. Specifically, we have removed statements implying a causal relationship between the B.1.1.413 variant and higher mortality during the third wave. Instead, we now emphasize that our epidemiological analysis revealed a higher mortality rate during this wave, and the median age of patients was indeed higher in the third wave compared to previous ones. As you astutely pointed out, variations in mean ages and outcomes among the waves introduce potential confounding factors that could influence the observed changes in hematological parameters. Therefore, we have reframed our discussion to acknowledge these limitations and have avoided drawing definitive conclusions regarding the cause of the observed differences in mortality rates. Instead, we have opted for a more cautious and data-driven approach, focusing on the description of the observed trends without speculative interpretations. Additionally, we have carefully incorporated all your suggested changes throughout the manuscript, figures, and tables. To ensure transparency and facilitate a comprehensive review, we have highlighted the modifications made in the revised version of the manuscript. By following your guidance, we believe our manuscript now offers a more accurate representation of our findings while ensuring a rigorous and unbiased scientific approach. We have also included a section in the revised manuscript that highlights the limitations of our study and the need for further research to investigate potential causative factors more comprehensively. We hope that you find the revised version more suitable and aligned with the rigorous standards of the journal.

**Q:** To the best of my understanding, the authors’ objective was to describe cohorts of COVID-19 patients using age, sex, disease outcome and hematological parameters during multiple waves of the pandemic. If this is the case, the current statistical analysis is appropriate. However, the authors should reconsider their phrasing to avoid making associations between specific waves and changes in hematological parameters throughout the manuscript.

**A:** We would like to express our gratitude for your thoughtful comment regarding the aim of our study. Your understanding aligns perfectly with our intentions for this research. Indeed, our primary objective was to describe cohorts of COVID-19 patients, analyzing age, gender, disease outcome, and hematological parameters across multiple waves of the pandemic. We acknowledge the importance of clarity in scientific writing, and your comment has prompted us to critically reconsider our phrasing throughout the manuscript. We understand that it is essential to avoid making unwarranted associations between specific waves and changes in hematological parameters, as this might inadvertently introduce bias or misinterpretation. In light of your valuable feedback, we have carefully revised the manuscript to ensure that our language emphasizes a descriptive approach rather than attempting to establish causative relationships or identify significant associations between specific waves and hematological changes. Our focus now remains on presenting the data and observations in a neutral and unbiased manner. We sincerely apologize for any ambiguity in our previous phrasing, and we thank you for bringing this to our attention. The revised version of the manuscript has been updated to reflect these improvements. Once again, we appreciate your diligence in reviewing our work and providing valuable suggestions that have undoubtedly strengthened the clarity and scientific rigor of our study.

**Q:** If the authors would like to make associations between their observations and specific waves of COVID-19, they should consider undertaking a matched analysis based on age, sex, disease outcome, and the severity of symptoms at time of hospital admission (if available). By matching patients with similar characteristics, the authors could control for potential confounding factors and improve the robustness of their analysis.

**A:** We would like to express our gratitude for your insightful comment regarding the potential associations between our observations and specific waves of COVID-19. Your suggestion to undertake a matched analysis based on age, sex, disease outcome, and symptom severity at the time of hospital admission is undoubtedly valuable for improving the robustness of our analysis. We have carefully considered your recommendation and understand the benefits of controlling for potential confounding factors. However, we wish to clarify our primary objective for this study. Our aim is not to establish causal relationships or make direct associations between specific waves of COVID-19 and changes in hematological parameters. Due to the limitations in our data and the retrospective nature of our study, conducting a matched analysis to control for potential confounders is challenging. Instead, we are primarily interested in exploring the epidemiological characteristics of COVID-19 patients, specifically focusing on age, sex, disease outcome, and hematological parameters, across multiple waves of the pandemic. By describing the cohorts of patients in each wave, we aim to identify trends and potential patterns in the data. We have thoroughly revised the manuscript to emphasize our intent and avoid any misinterpretation. We have carefully clarified throughout the text that our analysis does not permit making direct causal associations, and we now emphasize the descriptive nature of our study. We greatly appreciate your guidance in enhancing the clarity and scope of our research. Your thoughtful feedback has been invaluable in refining the manuscript to align more closely with our study's objectives and limitations.
